# Hyperosmolarity-induced AQP5 upregulation promotes inflammation and cell death via JNK1/2 Activation in human corneal epithelial cells

**DOI:** 10.1038/s41598-017-05145-y

**Published:** 2017-07-05

**Authors:** Yueping Ren, Huihui Lu, Peter S. Reinach, Qinxiang Zheng, Jinyang Li, Qiufan Tan, Hanlei Zhu, Wei Chen

**Affiliations:** 1 School of Ophthalmology and Optometry, Wenzhou Medical University, Zhejiang, China; 2 Jinhua Municipal Central Hospital, Zhejiang, China

## Abstract

Tear film hyperosmolarity and anterior ocular inflammation are two clinical signs that may be indicative of dry eye disease (DED). This condition can cause pathological and functional changes to the anterior ocular surface tissues. A contributing factor may be dysfunctional aquaporin 5 (AQP5) water channels as they are the AQP subtype that expressed in the corneal epithelium and contribute to fluid efflux needed for corneal function. We determined if described hyperosmolarity-induced increases in proinflammatory cytokine expression and cell death are mediated through AQP5 upregulation and JNK1/2 MAPK signaling activation in both primary human corneal epithelial cells (HCECs), and in a HCEC line. Real time RT-PCR identified rises in IL-1β, IL-6, IL-8, TNF-α, caspase-1, and AQP5 mRNA levels upon step increases in osmolarity up to 550 mOsm. Western blot analysis and the TUNEL assay identified corresponding rises in AQP5 and p-JNK1/2 protein expression and cell death respectively. JNK1/2 inhibition with SP600125, or siRNA AQP5 gene silencing reduced hypertonic-induced rises in proinflammatory cytokine expression and cell death. Taken together, hypertonicity-induced AQP5 upregulation leads to increases in proinflammatory cytokine expression and cell death through JNK1/2 MAPK activation. These results suggest that drug targeting AQP5 upregulation may be a therapeutic option in DED management.

## Introduction

Aquaporins (AQPs) are a family of different transmembrane spanning channel isoforms, which transfer water as well as small solutes and are expressed in bacteria, plants and animals. In mammalian tissues, at least 13 different AQP isoforms have been identified in mammals^1–3^.One of them is AQP5 which is expressed in human corneal epithelium and stromal keratocytes, while AQP1 is expressed in the corneal endothelium^4, 5^. AQP5 involvement in mediating fluid transport from the corneal stroma into the tears and contributing to maintaining tear film isotonicity is evident since in AQP5 knockout mice, tear fluid osmolality increased nearly two-fold^6^. Besides its involvement in offsetting osmotic stresses that disrupt tissue homeostasis, there is emerging evidence that AQP5 overexpression contributes to promoting increases in inflammation, cell proliferation, and migration in cervical, colon and gastric cancer^7–9^. Furthermore, AQP5 upregulation is associated with the development of airway inflammation and mucous hypersecretion during chronic asthma^10^.

Increases in tear film osmolarity promote transepithelial fluid efflux through epithelial AQP5 channels which allegedly dilutes the imposed stress and suppresses corneal epithelial volume shrinkage as well as declines in barrier function^11, 12^. However, in dry eye disease (DED) increases in water efflux may be inadequate through dysfunctional AQP5 channels, causing tear film osmolarity to remain invariant. Such constancy may cause increases in proinflammatory cytokine (interleukin (IL)-1β, tumor necrosis factor (TNF)- α, IL-6 and IL-8), chemokine and matrix metalloproteinases (MMPs) expression levels and cell apoptosis in corneal and conjunctival epithelial cells. Furthermore, exposure to hypertonic stresses induces immune cell activation and infiltration from the systemic circulation in different DED animal models^13–16^. Hypertonic-induced rises in fluid egress and AQP5 channel upregulation occur in some other tissues^17, 18^. However, it is unclear if AQP5 upregulation and MAPK signaling activation are also involved in mediating hypertonic-induced increases in proinflammatory cytokines and cell death in human corneal epithelial cells (HCECs).

AQP5 induced cell signaling pathway activation by hyperosmolarity has been characterized in some other tissues. In mouse lung epithelial cells, sequential increases in medium osmolality induced increases in AQP5 expression accompanied by ERK1/2 MAPK pathway activation^19^. On the other hand, in NIH3T3 cells, AQP5 overexpression stimulated proliferation through the Ras signal transduction pathway, which may account for the oncogenic properties described in AQP5 overexpressing cells^20^. Furthermore, modulation of AQP5 expression levels had corresponding effects on keratinocyte chemoattractant expression levels that were mediated through changes in ERK1/2 signaling activation^21^. Activation of the MAPK c-Jun N-terminal kinase (JNK) signaling pathway by conditions simulating those encountered in DED induced increases in production of MMP-1, MMP-9, and cornified envelope precursors^22, 23^. This pathway can also be activated through phosphorylation by stress stimuli such as tonicity, heat, or UV radiation, and affect changes in diverse cellular processes including cell apoptosis, proliferation, and survival^24, 25^.

We show here that hypertonic-induced increases in the expression levels of proinflammatory cytokines and cell death are associated with AQP5 upregulation and JNK1/2 pathway activation in primary cultures of HCECs as well as in a HCEC line. This association suggests that hypertonic-induced increases in AQP5 expression may contribute to offsetting the likelihood of increases in pathogenic infiltration resulting from declines in epithelial barrier function by inducing immune cell activation in DED.

## Results

### Hyperosmolarity dependent increases in AQP5 and proinflammatory cytokine expression

**Figure 1:**
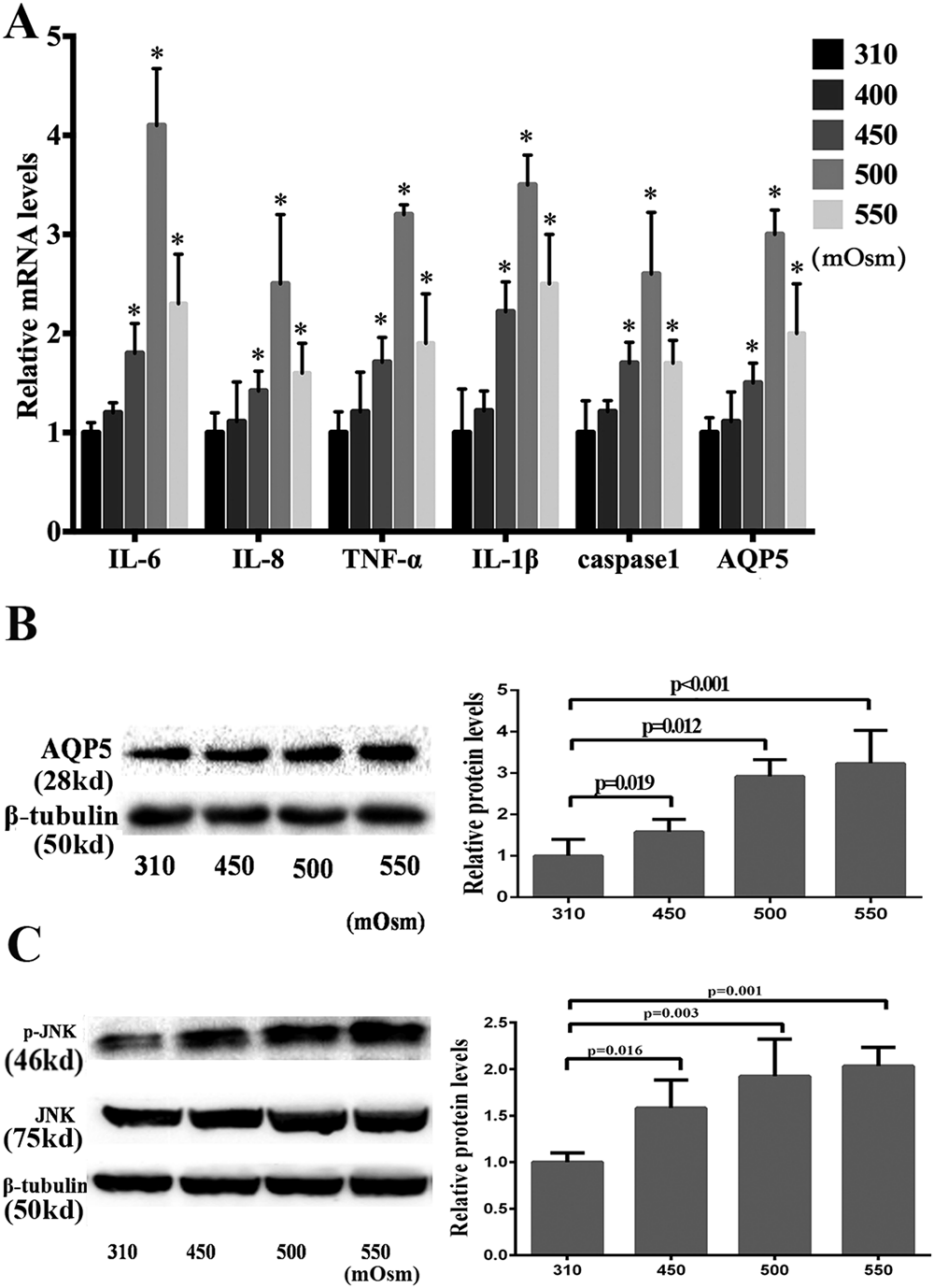
Differential effects of exposure to hyperosmolarity on proinflammatory cytokine and AQP5 expression levels in immortalized HCECs. The immortalized HCECs were cultured in media with varied osmolarities (310, 400, 450, 500 or 550 mOsm) for 4 h and 24 h respectively. The real time semi quantitative PCR analysis (4 h) showed the mRNA levels of IL-1β, IL-6, IL-8, TNF-α, an inflammatory mediator caspase-1, and AQP5 increased when the medium osmolarity increased to either 450, 500 or 550 mOsm relative to those in a 310 mOsm isotonic medium (*P* ≤ 0.034) (**A**). The protein expression levels measured by Western blot analysis of AQP5 (*P* ≤ 0.019) (**B**) and p-JNK1/2 (*P* ≤ 0.016) (**C**) rose after 24 h of exposure to hyperosmotic stress (450, 500 and 550 mOsm), while that of JNK remained unchanged in different groups. The cropped gels are displayed and the full-length gels are provided in a Supplementary Information file. Error bars represent the SD; * means *P* < 0.05 (Three independent experiments with 3 repeats each).

**Figure 2:**
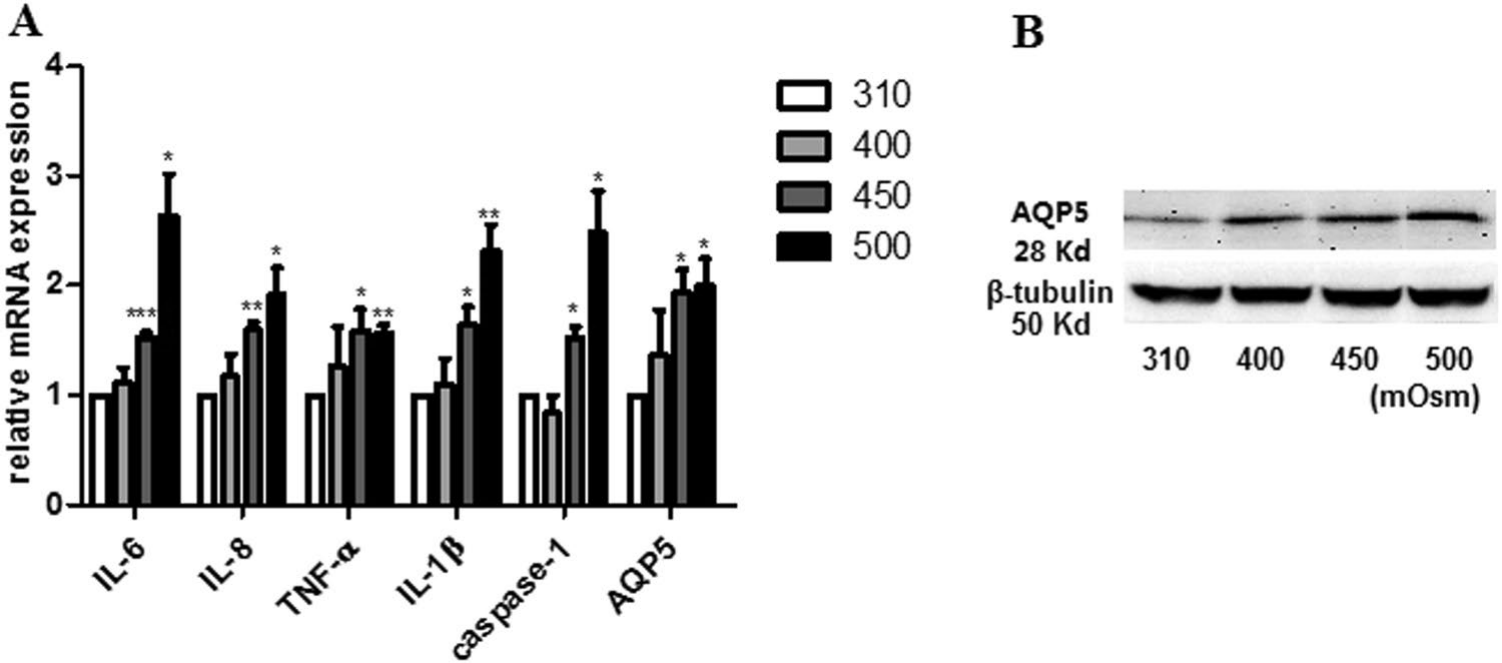
Validation of physiological relevance of results obtained with immortalized SV40-HCEC. Primary HCECs were cultured in media whose osmolarities were either 310, 400, 450 or 500 mOsm for 4 h and 12 h respectively. The mRNA levels (4 h) of IL-1β, IL-6, IL-8, TNF-α, caspase-1 and AQP5 marginally increased in those exposed to 400 mOsm group and significantly in those cultured in 450 and 500 mOsm (*P* ≤ 0.023), compared to the levels measured in isotonic 310 mOsm group (**A**). Western blot analysis (12 h) comfirmed that the AQP5 protein levels in the 400, 450 and 500 mOsm groups clearly increased over those in the isotonic 310 mOsm group (**B**). The cropped gels are displayed and the full-length gels are provided in a Supplementary Information file. Error bars representthe SD; * means *P* < 0.01; ** means *P* < 0.05; *** means *P* < 0.001 (Three independent experiments with 3 repeats each).

In immortalized HCECs, increasing their tissue culture osmolality to 400 mOsm for 4 h had no effect on the gene expression levels of IL-1β, IL-6, IL-8, TNF-α, interleukin-converting enzyme (ICE) or caspase-1, and AQP5. However, the results shown in Fig. 1A indicate that larger increases in medium tonicity to 450, 500 and 550 mOsm caused these levels to significantly rise further above those measured at lower levels (*P* ≤ 0.034). AQP5 and p-JNK1/2 protein expression levels rose after 24 h of exposure to either a hyperosmotic 450, 500 or 550 mOsm by at least by 50.2% (*P* ≤ 0.019) and 59.8% (*P* ≤ 0.016) respectively, while that of unphosphorylated JNK1/2 remained at levels similar to that in isotonic 310 mOsm (Fig. 1B,C). In order to confirm that the results are not cell-line dependent, we determined if such increases in gene and protein expressions levels can also occur in primary HCEC exposed to either 400, 450 or 500 mOsm for 4 h and 12 h, respectively. The mRNA levels of IL-1β, IL-6, IL-8, TNF-α, caspase-1 and AQP5 exhibited a trend to increase at 400 mOsm stress. Nevertheless, during exposure to either 450 or 500 mOsm these increases reached significance (*P* ≤ 0.023), compared to their levels in 310 mOsm (Fig. 2A). The rises in protein expression paralled those in gene expression occurring at 450 and 500 mOsm (Fig. 2B).

### AQP5 linkage to the JNK1/2 MAPK signaling pathway

**Figure 3:**
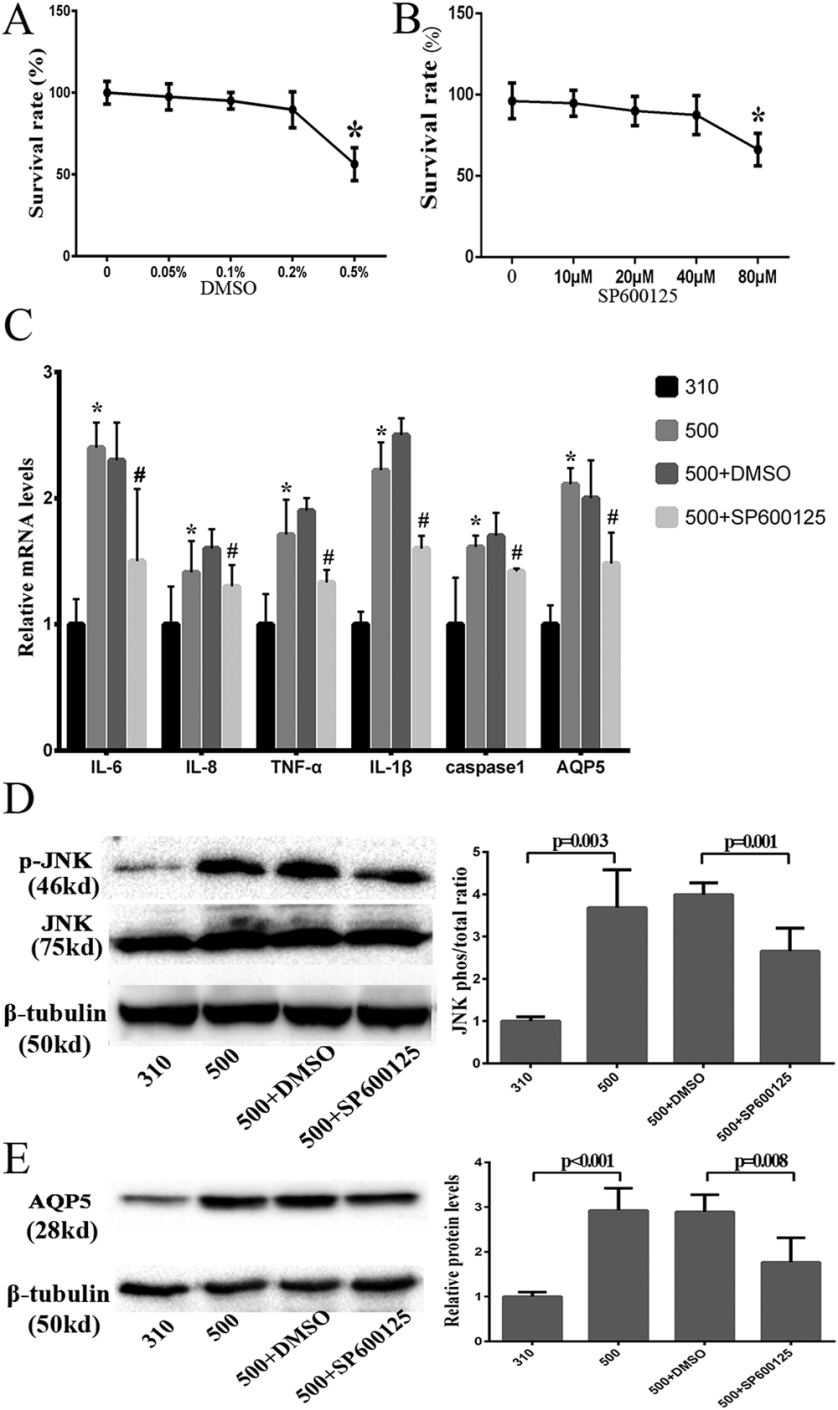
p-JNK1/2 inhibitor suppressed hyperosmolarity-induced AQP5 protein expression. The immortalized HCEC cell viability was relatively unchanged in the 0.05%, 0.1%, 0.2%DMSO groups but decreased significantly in the 0.5% DMSO group, compared to the control group (*P* = 0.018) (**A**). The cell viability in 10 μM, 20 μM, and 40 μM SP60012 dissolved in 0.1%DMSO was not different from that of the control group, while in the 80 μM SP600125 group cell viability significantly decreased (*P* = 0.037) (**B**). Pre-treatment with 20 μM SP600125 decreased IL-1β, IL-6, IL-8, TNF-α, caspase-1, and AQP5 mRNA levels in 500 mOsm medium during 4 h (*P* ≤ 0.012) (**C**), and the protein expression levels of p-JNK1//2 (**D**) and AQP5 (**E**) also declined by 33.3% (*P* = 0.001) and 39.3% (*P* = 0.008) respectively after 24 h. The cropped gels are displayed and the full-length gels are provided in a Supplementary Information file. Experiments were repeated three times each in triplicate.

**Figure 4:**
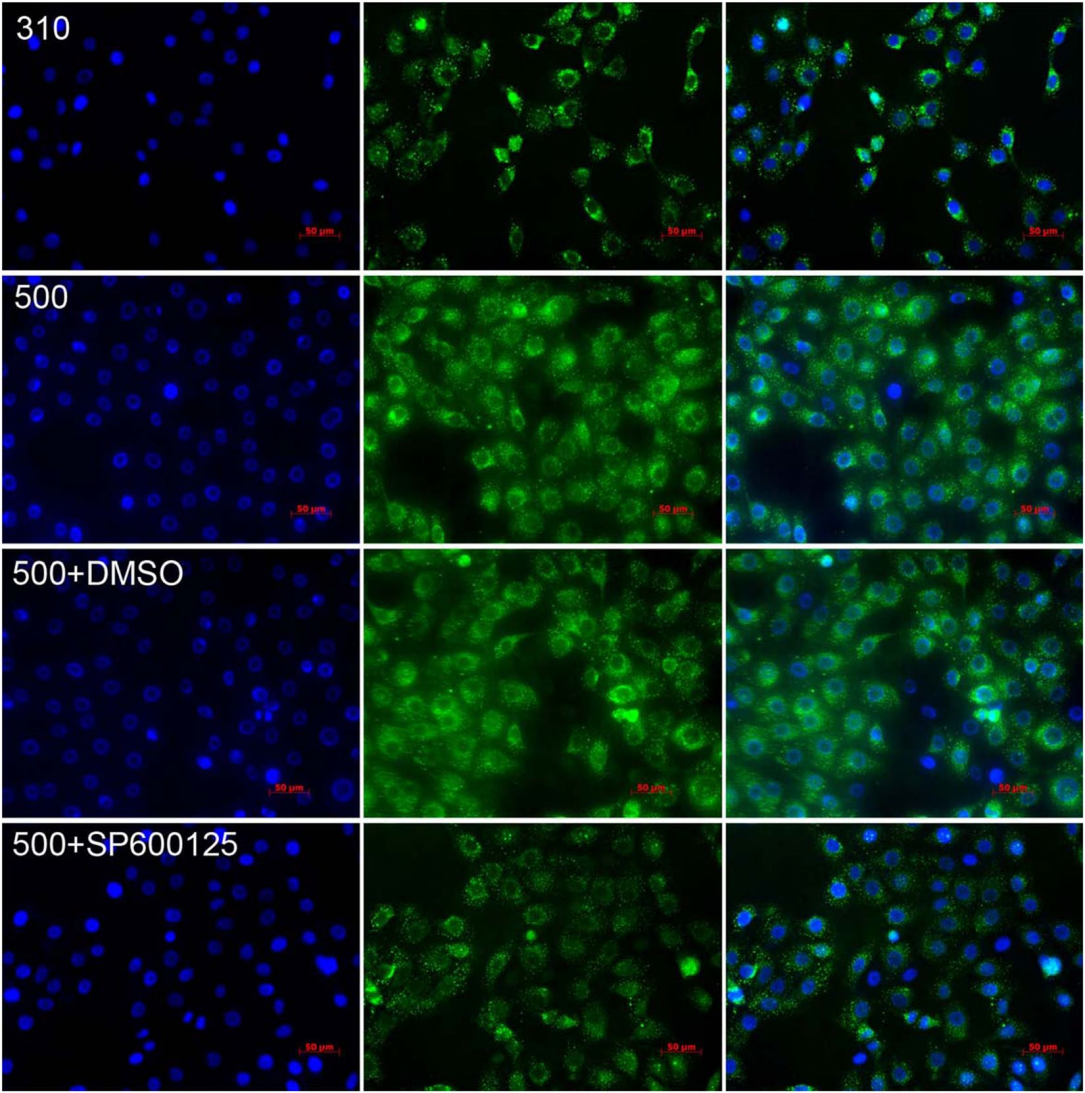
Effect of SP600125 on AQP5 protein levels determined by immunofluorescent localization. After 24 h in 500 mOsm medium, the immortalized HCECs were collected for immunofluorescent assay. AQP5 staining intensity distinctly declined with the pretreatment of 20 μM SP600125, confirming that JNK1/2 inhibition reduced rises in AQP5 expression levels induced by exposure to hyperosmotic 500 mOsm medium. DAPI (blue, the left column) shows the nuclei; FITC secondary antibody staining (green, the middle column) documents AQP5 expression; and the right column is the merged image. Experiments were repeated in three independent experiments. The top row: HCECs cultured in 310 mOsm; The second row from the top: HCECs cultured in 500 mOsm; The third row from the top: HCECs cultured in 500 mOsm and DMSO (the solvent of SP600125); The bottom row: HCECs cultured in 500 mOsm and 20 μM SP600125, a JNK inhibitor.

**Figure 5:**
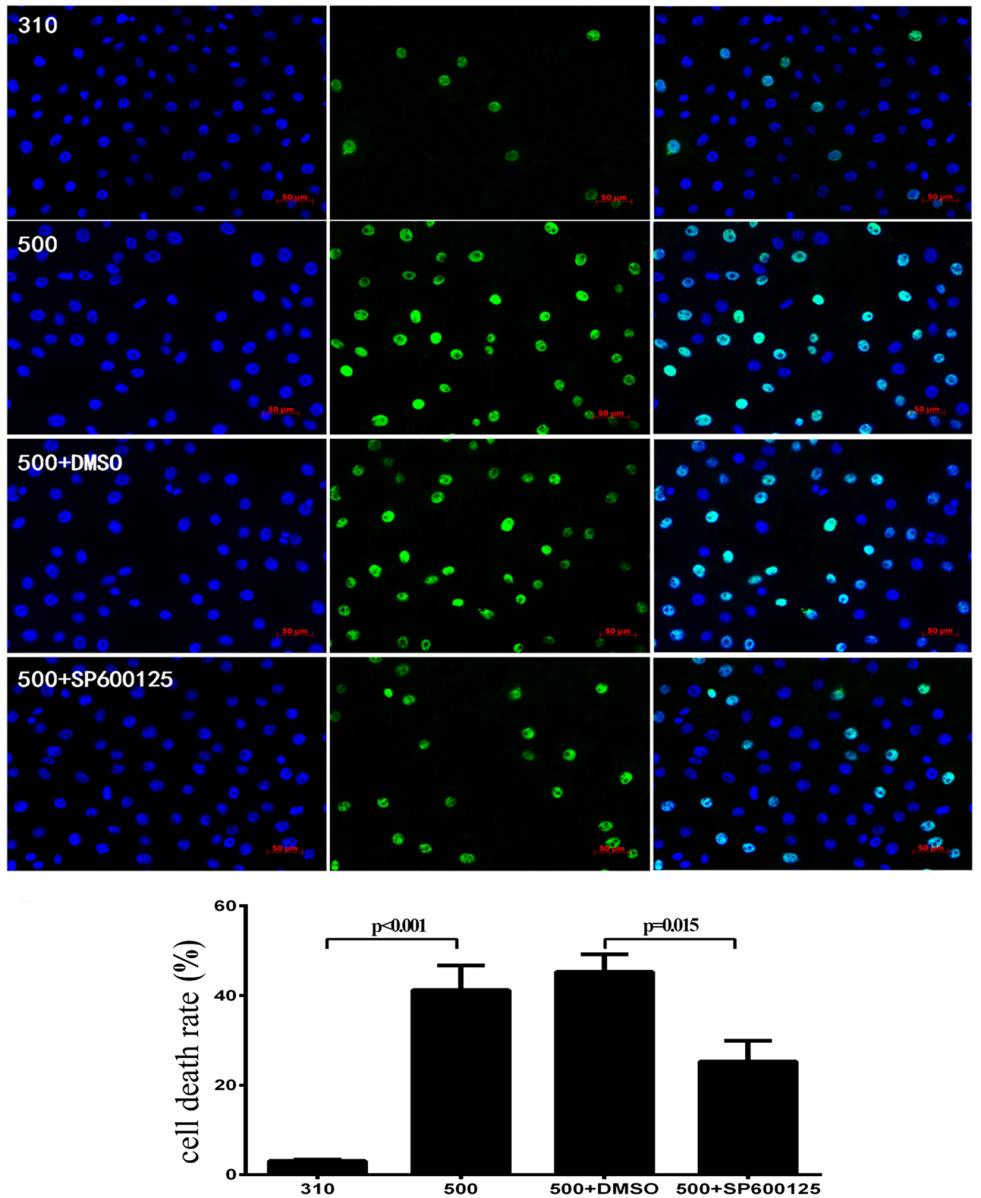
Effects of SP600125 on cell death. In immortalized HCECs, the TUNEL assay results show that cell death level increased in hyperosmotic 500 mOsm medium (*P* < 0.001), whereas SP600125 markedly reduced cell death staining by 44.6% (*P* = 0.015). DAPI (blue, the left column) shows the nuclei; FITC (green, the middle column) documents DNA fragmentation; and the right column is the merged image. Experiments were repeated three times each in triplicate.

The selective JNK1/2 pathway inhibitor, SP600125 was used to determine if hypertonicity-induced increases in AQP5 expression lead to rises in proinflammatory cytokine gene expression levels and cell death through stimulating the JNK1/2 MAPK signaling pathway in immortalized HCECs. Given the limited aqueous solubility of SP600125, we first identified a minimal working DMSO concentration which solubilized this drug without being cytotoxic. At a DMSO concentration as high as 0.2%, cell viability was retained whereas with 0.5% DMSO HCEC viability declined (*P* = 0.018) (Fig. 3A). Secondly, SP600125 up to 40 μM in 0.1% DMSO, was non-toxic whereas 80 μM was toxic (*P* = 0.037) (Fig. 3B) in the isosmotic medium. Accordingly, 20 μM SP600125 dissolved in 0.1% DMSO was selected to determine if AQP5 controlled proinflammatory cytokine and cell death levels through the JNK1/2 MAPK signaling pathway. During exposure to 20 μM SP600125, the mRNA levels of IL-1β, IL-6, IL-8, TNF-α, caspase-1, and AQP5 decreased significantly compared with those in the 500 mOsm + DMSO group (*P* ≤ 0.012) (Fig. 3C). AQP5 protein expression increased by 3.7-fold at 500 mOsm along with a 2.8-fold increase in p-JNK1/2 levels, whereas pretreatment with 20 μM SP600125 decreased the AQP5 and p-JNK1/2 protein expression by 33.3% (*P* = 0.001) and 39.3% (*P* = 0.008), respectively (Fig. 3D,E). To validate these aforementioned effects of hyperosmolar stress on AQP5 expression, *in-situ* immunofluorescence measurements were performed. With exposure to 500 mOsm medium, the increases in AQP5 staining intensity corresponded to the aforementioned increases in gene and protein expression. On the other hand, 20 μM SP600125 in a 500 mOsm medium dampened all of the increases that occurred during exposure to the 500 mOsm medium (Fig. 4). Regarding the effects of this hyperosmotic stress on cell death, TUNEL staining was also markedly reduced by 44.6% following exposure to 20 μM SP600125 (*P* = 0.015) (Fig. 5). This decline suggests that JNK1/2 MAPK inhibition provided a protective effect against hypertonicity-induced cell death.

### AQP5 siRNA knockdown suppresses hyperosmolarity-induced increases in proinflammatory cytokine expression and cell death

**Figure 6:**
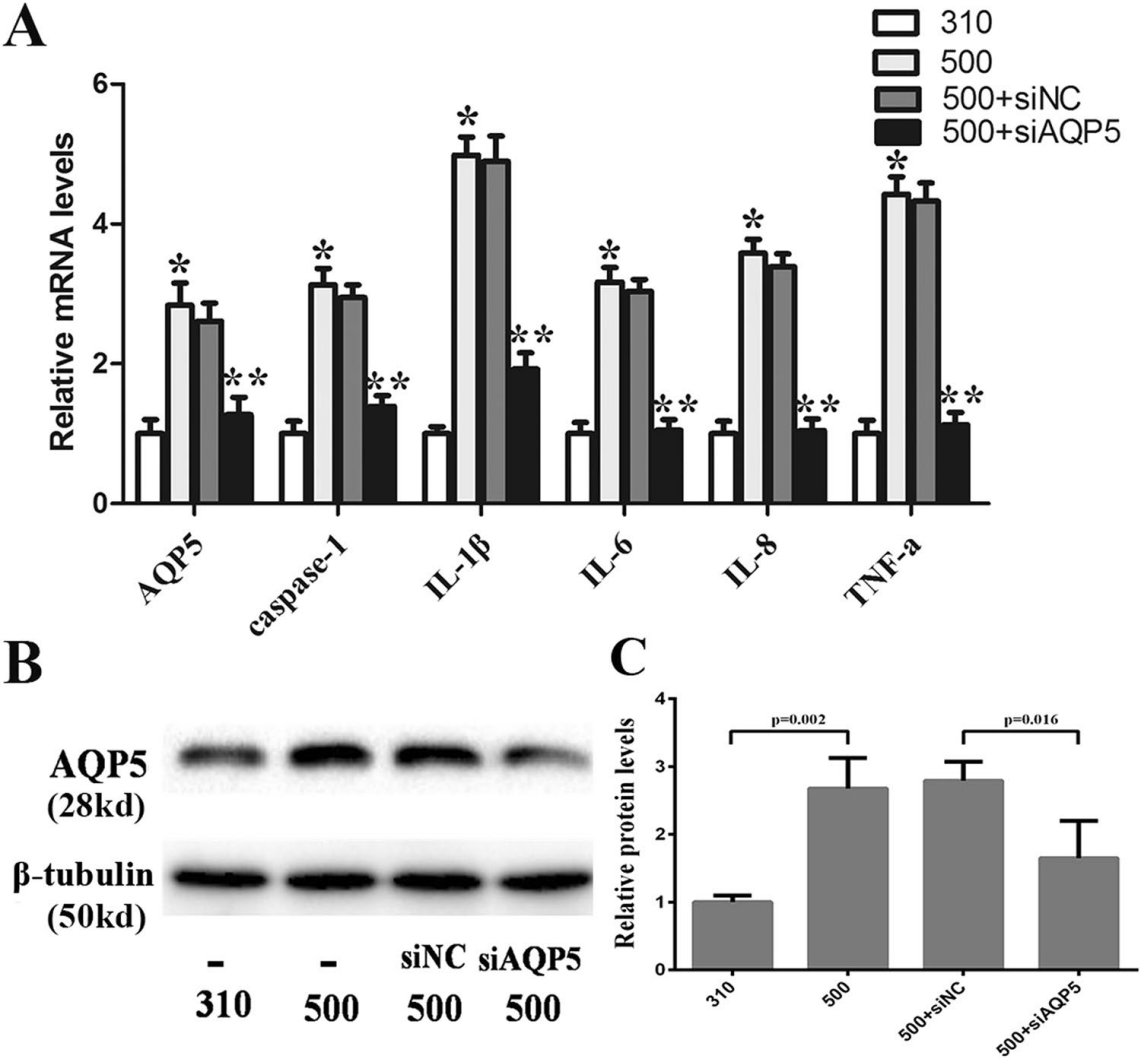
Effect of AQP5 gene silencing on hyperosmolarity-induced rises in proinflammatory cytokine expression levels. After AQP5 gene expression was silenced by targeted siRNA, the immortalized HCEC were cultured in 500 mOsm medium for 4 h (for RT-PCR) and 24 h (for western-blot). The mRNA levels of AQP5, IL-1β, IL-6, IL-8, TNF-α, and caspase-1 were significantly inhibited in the siAQP5 group compared with the siNC group (*P* ≤ 0.039) (**A**). Inhibition of AQP5 protein expression in AQP5 gene silenced cells confirms the targeted gene was efficiently knocked down (**B**). The cropped gels are displayed and the full-length gels are provided in a Supplementary Information file. * Indicates *P* < 0.05 in the comparsion between the 500 mOsm group and 310 mOsm group; and ** indicates *P* < 0.05 in the comparsion between the 500 mOsm + siAQP5 group and the 500 mOsm + siNC group.Three independent experiments were conducted with 3 repeats each.

**Figure 7:**
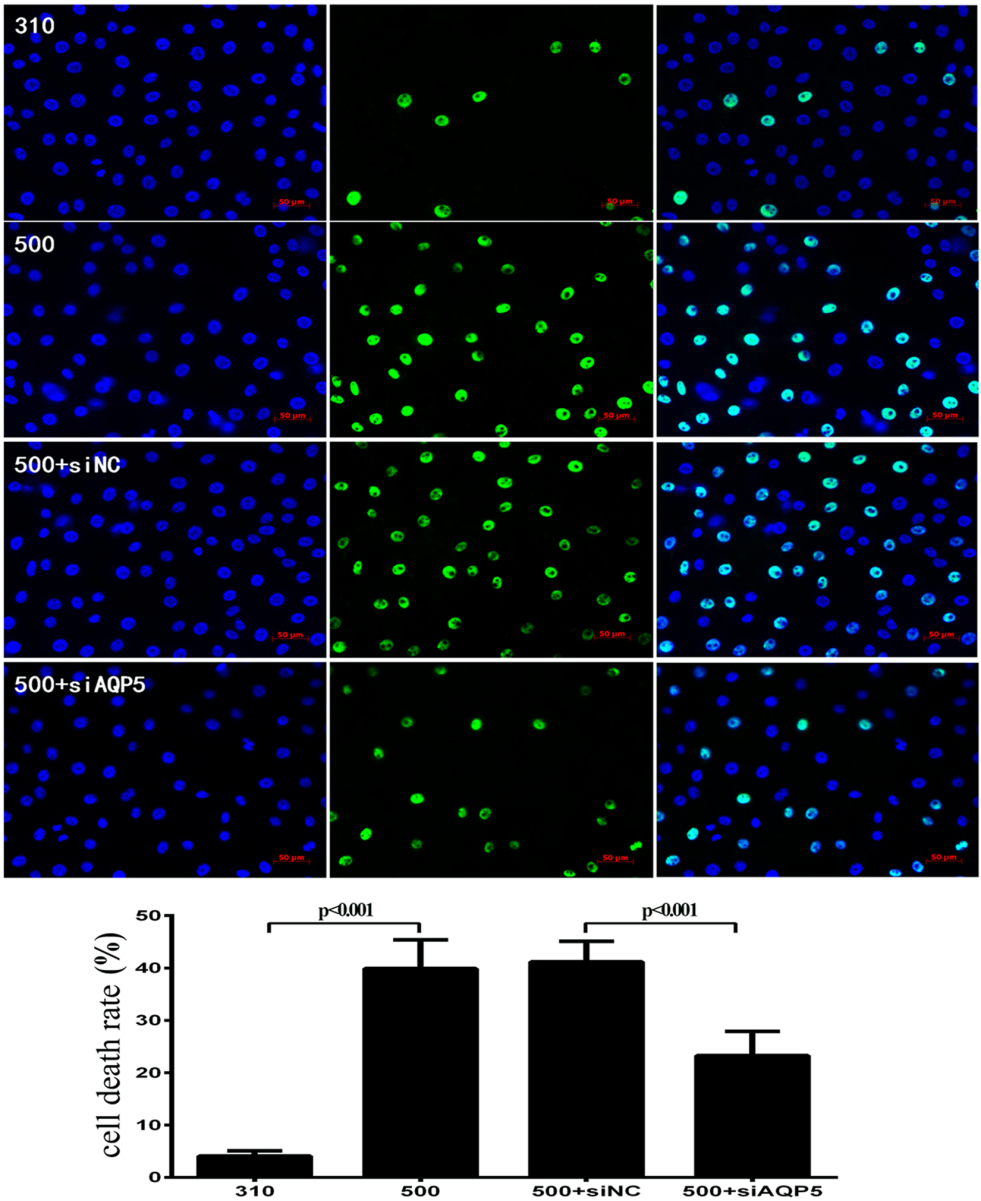
Effect of AQP5 gene silencing on hyperosmolarity induced rises in cell death. SiRNA AQP5 gene silencing in immortalized HCECs reduced hyperosmolarity-induced cell death. The level of cell death was reduced by 43.6% in siAQP5 group after exposure to 500 mOsm hyperosmotic medium for 24 h, compared to the cells in the siNC group (*P* < 0.001). DAPI (blue, the left column) shows the nuclei; FITC (green, the middle column) documents DNA fragmentation; and the right column is the merged image. Experiments were repeated three times each in triplicate.

Targeted siRNA AQP5 gene silencing down-regulated AQP5 gene and protein expression by 50.1% and 40.8%, respectively in immortalized HCECs (Fig. 6A,B,C). Following AQP5 knockdown and exposure to 500 mOsm, the mRNA levels of IL-1β, IL-6, IL-8, TNF-α, and caspase-1 were markedly lower than those in the siNCgroup (*P* ≤ 0.039) and comparable to those in the 310 mOsm group. The rises in all of these gene expression levels in the siNC group were indistinguishable from those in the non-siRNA transfected 500 mOsm group (Fig. 6A). However, the protein expression level of p-JNK1/2 was not different between the siAQP5 and siNC group in 500 mOsm medium (data not shown). On the other hand, cell death in the siRNA AQP5 silenced cells exposed to the 500 mOsm medium was 43.6% lower than that in the siNC group (*P* < 0.001) (Fig. 7).

## Discussion

We show here that hyperosmotic media-induced increases in AQP5 and p-JNK1/2 protein expression are accompanied by rises in proinflammatory cytokine gene expression and cell death in HCECs. This rise in AQP5 protein expression is dependent on JNK1/2 activation since SP600125 blocked all of these responses. Another indication of the involvement of AQP5 in inducing these hyperosmotic-induced responses is that AQP5 gene silencing blunted these increases along with augmentation of cell death without altering the p-JNK1/2 protein expression level.

AQP channel mediated water permeation across cell membranes provides an essential route for rapid transcellular water flow induced by an anisosmotic stress^26^. Such responses are crucial for restoring intracellular isotonicity during exposure to stresses that activate cell volume regulatory behavior^27^. There are a number of reports showing that AQP isoforms are essential to maintaining a number of different ocular functions. In different tissues, loss of AQP function caused losses in corneal and lens transparency, increases in intraocular pressure and altered retinal signal transduction^28, 29^. AQP5 is the major AQP isoform expressed in the human corneal epithelium and it contributes to adapting to imposed osmotic challenges by increasing water permeation and hastening restoration of intracellular isotonicity. Its involvement in this process is evident based on showing that during exposing AQP5 knockout mice to a hypotonic tear-side bath their corneal epithelial thickness increased 17% more than their wildtype counterpart even though the rate of thickening in the AQP5 knockout group was about 46% slower than that in their wildtype counterpart^30^. This difference in restorative capacity between these two groups provides insight into the contribution made by AQP5 in offsetting the natural tendency of the cornea to imbibe fluid and cause corneal swelling which can lead to losses in transparency if the thickness increases by more than 20%^31^. Even though it is frequently shown that induced increases in protein expression are reflective of an increase in functional activity, it is unclear from the current study if AQP5 upregulation is indicative of such an effect. This uncertainty exists because an adaptive response to a hypertonic challenge is expected to involve a decline in osmotic-driven water efflux. However, in DED despite increases in AQP5 expression fluid efflux is impaired through the membrane bilayer and constituent AQP5 channels. This consideration suggests that the hypertonic-induced increase in AQP5 expression may be unrelated to a presumed adaptive decline in membrane water permeability induced by a hyperosmotic stress.

Hypertonicity directly controls the expression levels of AQPs in different cell types^32, 33^. One possibility is that such regulation is mediated through the tonicity response enhancer element (TonE)^34, 35^. Matsuzaki *et al*. showed that a tonicity-responsive region colocalizes with the classical TonE in the AQP2 gene. Furthermore, the hypertonicity-induced activity of AQP2 was stimulated by the overexpression of TonE-binding protein (TonEBP), indicating that TonE is responsible for hypertonic regulation of AQP-2 transcription^35^. They further showed that hypotonicity decreases the AQP-2 promoter activity mediated via TonE by activating JNK1/2^34^.

The involvement of AQPs including AQP5 in mediating hypertonic-induced inflammatory responses was demonstrated in different tissues, in which the downregulation of their expression significantly reduced IL-1β release and immune cell infiltration^36–40^. Moreover, inhibition of AQPs in various cultured cells, not only blocked the apoptotic volume decrease (AVD) occurring during cell apoptosis, but it also prevented downstream apoptotic events including cytochrome c release, caspase-3 activation, and DNA degradation^41, 42^, which suggests that AQP-mediated cell volume shrinkage plays an essential role in both the initiation and completion of apoptosis. Subsequent to AVD termination, AQP facilitation of water efflux declined while K^+^ efflux was unchanged, resulting in declines in intracellular K^+^ activity which activated apoptotic enzymes and apoptosis^43^. Our results are consistent with these other reports in that hyperosmolarity stress increased AQP5 expression levels whereas AQP5 siRNA gene silencing led to declines in hypertonic-induced increases in proinflammatory cytokine expression as well as cell death in HCECs.

The JNK1/2 MAPK signaling pathway, also named the stress-activated protein kinase pathway (SAPK), is essential for mediating cellular responses to various stimuli. Some of stresses known to induce increases in the JNK1/2 phosphorylation status include, exposure to reactive oxygen species (ROS), mechanical shear stress and a hyperosmolar challenge^13, 44, 45^. *In vitro* and *in vivo* results showed that LPS decreases AQP5 expression through the p-JNK/NF-κβ signaling pathway^46, 47^. On the other hand, JNK1/2 inhibition suppressed AQP1, MMP-9, and caspase-3 activation, leading to reduced brain injury in rats^48^. Our results show that JNK1/2 inhibition with SP600125 reduced AQP5, IL-1β, IL-6, IL-8, TNF-α and caspase-1 expression and cell death, while AQP5 gene silencing had no effect on the p-JNK1/2 expression level. These results indicate that the hypertonic-induced rises in AQP5 protein expression may be regulated by JNK1/2 activation. It is also possible that 50% knockdown of AQP5 did not suppress p-JNK1/2 formation because the remaining active 50% AQP5 may still be sufficient to similarly activate p-JNK1/2 formation. Additional studies are needed to determine if AQP5 is an appropriate drug target in some types of dry eye disease because hyperosmotic-driven increases in fluid egress through AQP5 channels contribute to offsetting such stress. This dependence on AQP5 upregulation is apparent because tear film osmolarity and presumably corneal thickness increased more in AQP5 knockout mice than in their wildtype counterpart^30^. As already pointed out, it may be possible to better assess if AQP5 is a viable drug target to offset hypertonic induced increases in proinflammatory cytokine expression and cell death by determining in a future study if increases in AQP5 gene and protein expression are indeed indicative of increases in its water permeability.

In spite of numerous reports indicating a relationship between tear film hyperosmolarity and ocular inflammation in dry eye patients^49–51^, the physiological relevance of the findings in this study awaits future clarification. Such uncertainty exists because the tear film osmolarity measurements of most dry eye patients are lower than those needed to induce rises in HCEC AQP5 expression and proinflammatory cytokine secretion along with cell death in this study. However, the actual localized tear film osmolarity inducing inflammation and cell death in these individuals is open to question because there is speculation that its value may fluctuate depending on blink frequency and vary because of tonicity compartamentalization over the ocular surface^52^.

Taken together, exposure to a hyperosmotic challenge induces AQP5 upregulation along with increases in expression levels of proinflammatory cytokine as well as an inflammatory mediator caspase-1 and cell death through increases in the JNK1/2 MAPK signaling pathway in HCECs. It is conceivable that effective agents will be developed that overcome dysfunctional AQP5 involvement in DED through stimulating rather than inhibiting its activity without increasing its gene and protein expression levels.

## Material and Methods

### Cell culture and SP600125 treatment

The human SV40 immortalized corneal epithelial cell line (CRL-11135, HCE-2; ATCC, Manassas, VA), between passages 60 and 65, was cultured in DMEM/F12 with 5% FBS and 10 ng/ml human epidermal growth factor (Invitrogen-Gibco, Gaithersburg, MD) and the medium was replaced every other day. Subconfluent (70–80%) cultures were switched to a serum-free medium (DMEM without FBS) for 24 h before treatment. Then the cells were cultured for an additional 4 or 24 h in an equal volume (1.0 ml/well) of serum-free medium whose osmolarities were either 310, 400, 450, 500 or 550 mOsm obtained by adding 0, 50, 70, 90 or 120 mM sodium chloride (NaCl). Cell cultures in 500 mOsm media were pretreated with or without a JNK inhibitor, 20 µM SP600125 (Sigma-AldrichInc., St. Louis, MO, US), which was added 2 h before NaCl supplementation. Cells collected at 4 h were stored for realtime PCR analysis. AQP5 expression levels were measured following 24 h incubation with an immunofluorescent assay, and western blot analysis whereas the latter method evaluated changes in JNK1/2 phosphorylation status. The TUNEL assay measured cell death. In order to validate that the results are not cell-line dependent, the primary HCECs were cultured in 310, 400, 450, and 500 mOsm media. Cells collected at 4 h and 12 h were used for realtime PCR and western blot analysis, respectively. However, the primary HCECs were only used in the experiments to evaluate the hyperosmolarity-stressed mRNA levels of IL-1β, IL-6, IL-8, TNF-α, caspase-1 and AQP5, as well as the western blot results of AQP5 protein. Human corneoscleral tissues not qualified forclinical use from donors aged 20 to 50 years were obtained from the Wenzhou Eye Bank of Zhejiang province, China. The primary HCECs were grown from limbal explants using the same method described by Li *et al*.^53^. Written informed consents declaring that the donor eyeballs were used for clinical patients or scientific research were obtained from all the donors or their guardians (Donor number: WZEB786–809). And this study was approved by the Institutional Review Board of Wenzhou Medical University. We declare that all methods used in the current study were performed in accordance with the guidelines and regulations required by the Institutional Review Board of Wenzhou Medical University.

### RNA interference

SV40 HCECs were transfected at 70% confluence using Lipojet transfection reagent (SignaGen, Rockville, MD), and siRNA against AQP5 (sense, 5′-GCGUGUGGCCAUCAUCAAATT-3′, and antisense, 5′-UUUGAUGAUGGCCACACGCTT-3′) or a physiologically irrelevant negative control (NC) siRNA (sense, 5′-UUCUCCGAACGUGUCACGUTT-3′, and antisense, 5′-ACGUGACACGUUCGGAGAATT-3′). Each dried-down siRNA was dissolved in nuclease-free water to achieve a final concentration of 20 μM. Then 2 ml of this solution and 2 ml Lipofectamine 2000 were added to a 100 ml buffer system. The mixes were kept at room temperature for 10–15 min to form complexes, and equal aliquots were then added into one of the wells of a 6-well plate. The cultures were incubated at 37 °C in a 5% CO_2_ incubator. The medium was replaced after 24 h with either 310 or 500 mOsm medium that did not contain either a siRNA or the transfection reagent. Cells were collected at 4 or 24 h for mRNA or protein expression analyses, respectively.

### Real-time PCR

Total RNA was extracted from collected cells (RNA lysis buffer RLT; Applied Biosystems, Grand Island, NY) and 0.2 mg of RNA from each sample was reverse transcribed with M-MLV reverse transcriptase (Applied Biosystems) according to the manufacturer’s instructions. The sequences of the primers were: for AQP5: sense, 5′-GCTCACTGGGTTTTCTGGGTA-3′, and antisense, 5′-TCCATGGTCTTCTTCCGCTC-3′; for IL-1b: sense, 5′-AGCTACGAATCTCCGACCAC-3′, and antisense, 5′-CGTTATCCCATGTGTCGAAGAA-3′; for IL-6: sense, 5′-AAATCACCATGCACCTCATCC-3′, and antisense, 5′-AGAGGATTGTGCCCGAACTAAA-3′; for IL-8: sense, 5′-ATGCTTTTGATCTGCACAGCTGCAC-3′, and antisense, 5′-TGGTCCAGCAGGAATAACCCTCAG-3′; for TNF-α: sense, 5′-CAGCCTCTTCTCCTTCCTGA-3′, and antisense, 5′-GGAAGACCCCTCCCAGATAGA-3′; for Caspase-1: sense, 5′-TTTCCGCAAGGTTCGATTTTCA-3′, and antisense, 5′-GGCATCTGCGCTCTACCATC-3′; for GAPDH: sense, 5′-ATGTTCGTCATGGGTGTGAA-3′, and antisense, 5′-GGTGCTAAGCAGTTGGTGGT-3′. PCRs were performed using a 7500 Real-Time PCR System (Applied Biosystems), with 2“SYBR® Green PCR Master Mix (Applied Biosystems). The results were normalized to GAPDH.

### Western blotting

Protein was extracted using ice-cold lysis buffer and ultrasonic dispersion. The suspension was centrifuged at 15,000 rpm for 15 min and the supernatant protein content was determined with the BCA assay. Samples containing 30 µg protein were subjected to SDS-PAGE and then transferred to polyvinylidene difluoride (PVDF) membranes (Roche, Switzerland). Membranes were blocked with fat-free milk and then probed overnight with primary anti-AQP5 (sc-28628, Santa Cruz Biotechnology, Santa Cruz, CA, 1:500) primary anti-JNK (ab179461, Abcam, MA, USA, 1:500) and primary anti-p-JNK1/2 (ab124956, Abcam, MA, USA, 1:500), followed by incubating with horse radish peroxidase (HRP) conjugated goat anti-rabbit IgG (Bioworld technology, Nanjing, China, 1:5000). Then they were developed with an an ECL detection system (Santa Cruz Biotechnology, Inc., Santa Cruz, CA, USA). β-tubulin was used as a loading control.

### Immunofluorescent assay

SV40 HCECs were plated on 12-well plates (Corning, USA) in complete medium at 37 °C with 5% CO_2_. Cells were fixed with 4% paraformaldehyde for 30 min at room temperature, blocked in 10% serum at 37 °C for 30 min, and finally incubated with the AQP5 antibody (sc-28628, Santa Cruz Biotechnology, Santa Cruz, CA, 1:200) at 4 °C overnight. Then the cells were incubated with a matched Alexa Fluor 488-conjugated secondary antibody (Thermo Fisher Scientific, USA, 1:200) and DAPI (Beyotime Biotechnology, Shanghai, China, 1:1000) for 15 min in dark at room temperature and visualized and recorded using fluorescence microscopy (Zeiss, Japan).

### TUNEL assay

The SV40 cell activity was measured by terminal-deoxynucleoitidyl transferase mediated nick end labeling (TUNEL) kits (Roche, Switzerland). TUNEL was performed as described in the instructions provided by the manufacturer.

### Statistical analysis

SPSS 18.0 (IBM Corporation, Armonk, NY) evaluated significance and GraphPad Prism 6 (GraphPad Software Inc., La Jolla, CA) was used to generate figures and tables. Significance between two groups was evaluated using nonpaired Students-T-test. One-way ANOVA with Bonferroni correction was used for comparison between three or more groups. The correlation between two parameters was analyzed by Pearson’s correlation and linear regression. Differences were defined as significant at *P* < 0.05. All values are expressed as means ± SD (Standard Deviation).

## Electronic supplementary material

Supplementary Information
