# Supplementary material for: Hyperosmolarity-induced AQP5 upregulation promotes inflammation and cell death via JNK1/2 Activation in human corneal epithelial cells
Source: Sci Rep. 2017 Jul 5;7:4727. doi: 10.1038/s41598-017-05145-y (PMC5498491; doi:10.1038/s41598-017-05145-y)
Supplement: Supplementary file 1 — Supplementary Information [file 41598_2017_5145_MOESM1_ESM.pdf]

Hyperosmolarity-induced AQP5 upregulation promotes inflammation and cell death via JNK1/2 Activation in human corneal epithelial cells

Yueping Ren[1], Huihui Lu[2], Peter S. Reinach[1], Qinxiang Zheng[1], Jinyang Li[1], Qiufan Tan[1], Hanlei Zhu[1] and Wei Chen[1]*

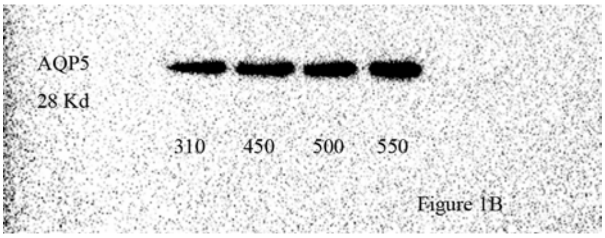

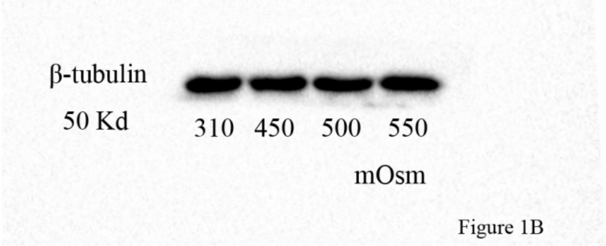

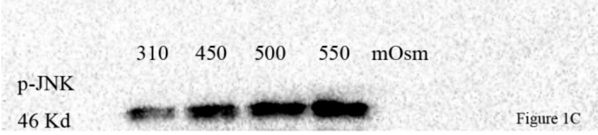

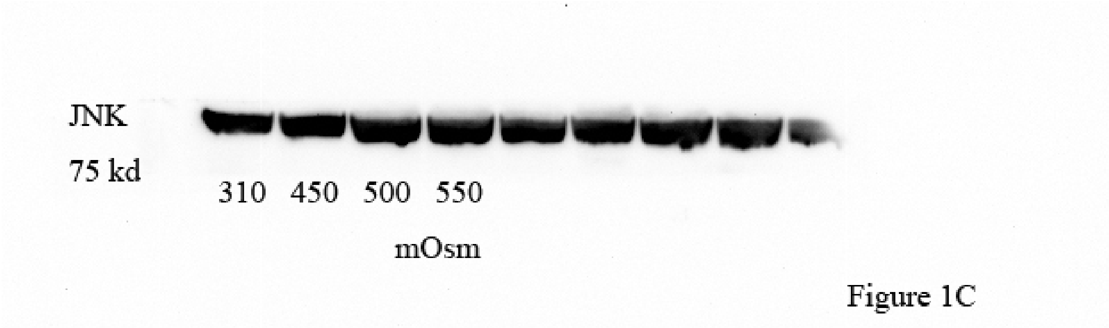

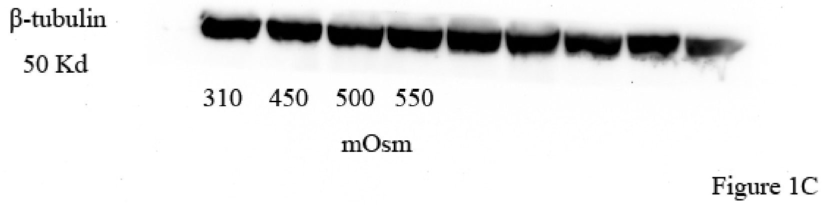

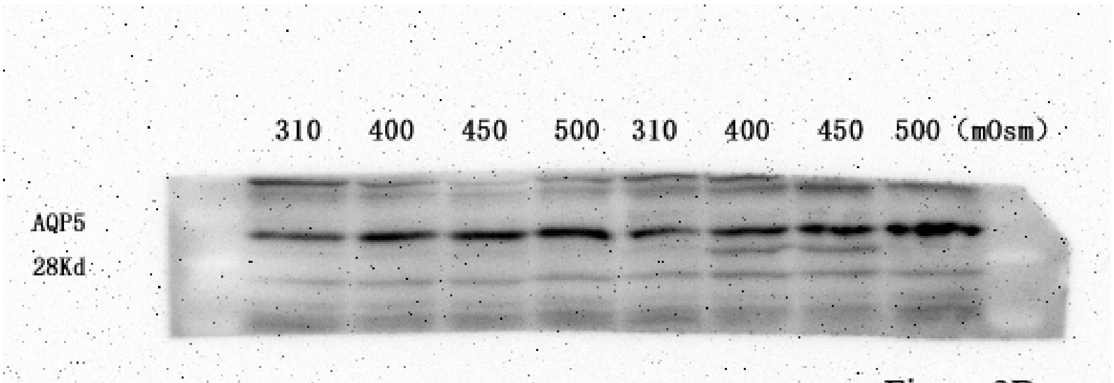

Figure 2B

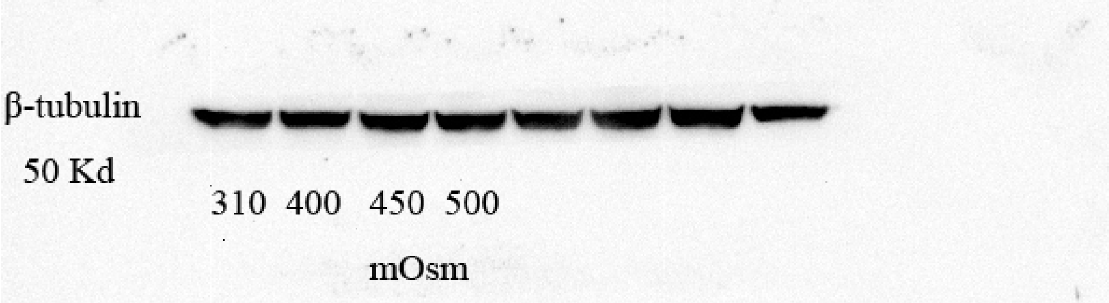

Figure 2B

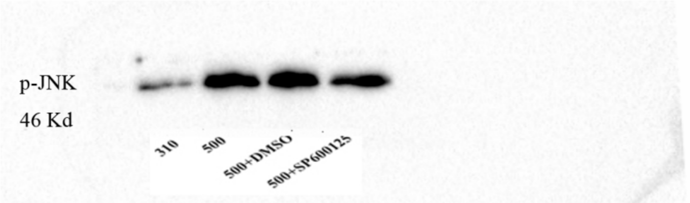

Figure 3D

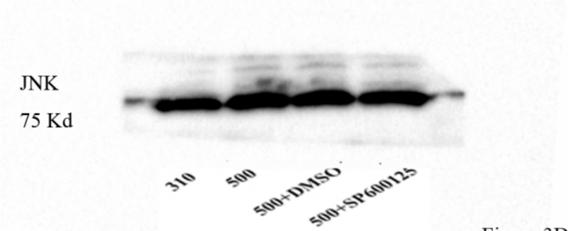

Figure 3D

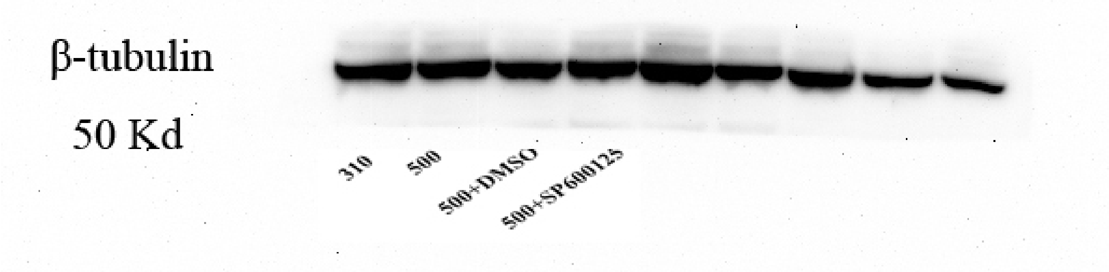

Figure 3D

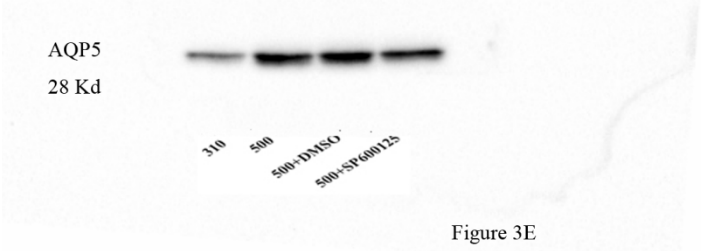

Figure 3E

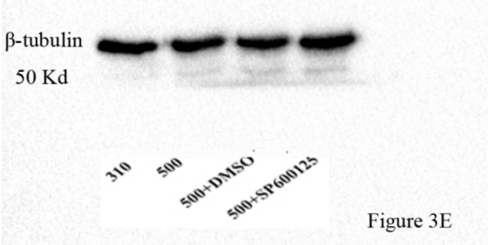

Figure 3E

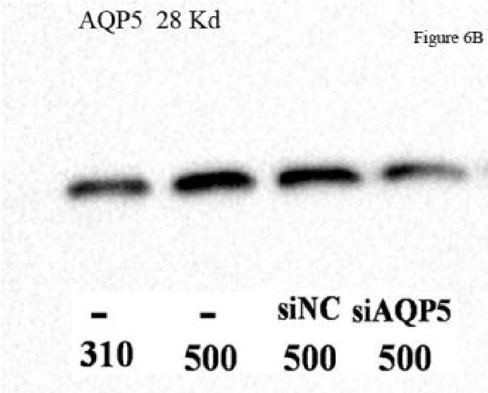

Figure 6B

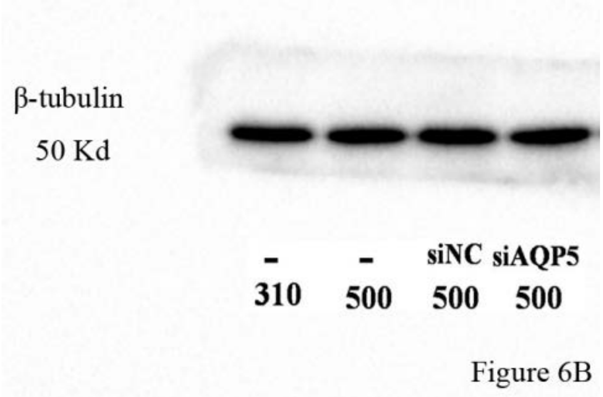

Figure 6B
